# Occurrence, Antimicrobial Resistance, and Virulence Profiles of *Salmonella* Serovars Isolated from Wild Reptiles in South Africa

**DOI:** 10.1155/2024/5213895

**Published:** 2024-01-05

**Authors:** Lungile N. Mlangeni, Tsepo Ramatla, Kgaugelo E. Lekota, Cormac Price, Oriel Thekisoe, Che Weldon

**Affiliations:** ^1^Unit for Environmental Sciences and Management, North-West University, Potchefstroom 2531, South Africa; ^2^Gastrointestinal Research Unit, Department of Surgery, School of Clinical Medicine, University of the Free State, Bloemfontein 9300, South Africa

## Abstract

Reptiles are carriers of an array of microorganisms, including significant zoonotic bacteria of the genus *Salmonella*, which cause a disease referred to as salmonellosis that affects both animals and humans. This study investigated the occurrence of *Salmonella* serovars in wild reptiles at Timbavati Private Game Reserve in Limpopo Province, South Africa, and examined their virulence and antimicrobial resistance gene profiles. A total of 19 wild reptiles were sampled, which resulted in 30 presumptive *Salmonella* isolates. The isolates were identified using polymerase chain reaction (PCR) by amplifying the *invA* gene and were further confirmed by *16S* rRNA gene sequencing. *Salmonella* serovars were detected in chameleons (36.8%), lizards (31.6%), snakes (15.8%), and tortoises (15.8%). The use of 16S rRNA gene sequencing revealed that *Salmonella enterica* subsp. *enterica* serovar Salamae (30%), *S*. *enterica* subsp. *enterica* (16.7%), *S*. *enterica* subsp. *enterica* serovar Typhimurium (13.3%), and *S*. *enterica* subsp. *enterica* serovar Indiana (13.3%) were the four most common subspecies among the investigated 30 isolates. Detected virulence genes included *pag*N (100%), *hil*A (96.7%), *ssr*B (96.7%), *prg*H (86.7%), and *mar*T (86.7%). The isolates exhibited resistance to nalidixic acid (43.3%) and kanamycin (43.3%), followed by streptomycin (16.7%) and ciprofloxacin (3.3%). Antibiotic-resistant genes were detected as follows: *strA*, *strB*, *qnrA*, *qnrS*, *parC*, *aadA*, *aac*(*6*′)-*Ib*, and *aac*(*6*′)*-Ib-cr* at 33.3%, 6.7%, 16.7, 13.3%, 10%, 23.3%, 6.7%, and 10%, respectively. The findings highlight the necessity of educational initiatives aimed at reducing reptile-related infections. Effective antibiotic treatment appears promising for infection, given the minimal drug resistance observed in reptile *Salmonella* serovars in the current study.

## 1. Introduction

*Salmonella* is a genus that belongs to the family *Enterobacteriaceae*, a Gram-negative facultative anaerobic bacterium, and is regarded as one of the most concerning zoonotic bacteria in the world [1, 2]. *Salmonella* is naturally present in the gastrointestinal tracts of many species of animals, including humans, birds, reptiles, and livestock [3, 4]. The species *S. enterica* is comprised of six subspecies: indica, salamae, enterica, houtenae, arizonae, and diarizonae. It is estimated to have more than 2659 serovars, which are divided into 60 serogroups [5, 6]. According to the current nomenclature, *Salmonella* spp. is taxonomically classified into two species: *S. bongori* and *S. enterica* [7]. *Salmonella* is generally considered a normal constituent of the reptilian intestinal microbiota with a subclinical presentation [1]. Nevertheless, some reptiles harbor and shed *Salmonella* spp. asymptomatically in their faeces, and up to 90% of them are considered reservoirs for the bacteria [8]. In South Africa, *Salmonella* serovars have previously been documented in farmed crocodiles and a few other, mostly captive reptiles [9]. However, the association of reptile-associated *Salmonella* in South Africa is largely unknown.

There have even been several outbreaks of human salmonellosis associated with reptiles from various countries [8, 10, 11]. Assessing the risk of humans being infected through direct contact with reptiles becomes challenging due to the lack of a robust understanding of the natural occurrence of *Salmonella* spp. circulating in reptiles and their propensity to switch hosts [1]. The risk of zoonotic disease is higher with the transmission of multidrug-resistant *Salmonella* spp. strains. The presence of plasmids, transposons, integrons, and insertion sequences can contribute to the development of antibiotic resistance [12, 13]. There have been numerous studies on antibiotic resistance genes identified in *Salmonella* spp. [12–14]. Most virulence and resistance genes have been transferred between species by horizontal gene transfer (HGT) [15]. Virulence plasmids, pili, and enterotoxins are among the reported *Salmonella* pathogenicity islands (SPIs) [16]. Virulence mechanisms are required to defeat host defense systems, and the development of antimicrobial resistance is required to allow pathogenic bacteria to overcome antimicrobial therapy and adapt to and thrive in competitive and demanding environments [15, 17, 18]. The virulence genes contribute to pathogenesis through host cell attachment and overcoming host defense mechanisms [14]. Infection and virulence are often associated with antibiotic resistance, as seen in biofilm-producing bacteria or intracellular infections [15, 16]. Therefore, the aim of this study was to determine the prevalence of *Salmonella* spp. in various wild reptile species and to evaluate their antimicrobial resistance and virulence gene profiles. The remarkable array of reptile diversity in this region acts as a catalyst for the exploration of antibiotic resistance, with ultimate benefits for reptile conservation.

## 2. Materials and Methods

### 2.1. Field Site

The Timbavati Private Game Reserve is situated between 24°24′S and 31°21′E. It covers an area of 550 km^2^ and is located on the central west border of Kruger National Park. The reserve comprises *Combretum apiculatum*, *Acacia nigrescens*, and *Colophospermum mopane* as the dominant vegetation types, with mostly granite or basalt as the principal soil types [19].

### 2.2. Collection of Samples

Samples were collected from wild reptiles (*n* = 19) in the Timbavati Private Game Reserve in the Limpopo Province. Collection consisted of active searching for wild reptiles and their subsequent release after sampling. Snakes were placed in transparent plastic tubes before sampling, while other reptiles were restrained by hand [20]. Sterile cotton transport swabs (Transystem™) were used to swab the cloaca of the reptiles and were stored at 4°C during field work [21]. The transport medium provides a nonnutritive environment that maintains the viability of microorganisms while restricting growth until samples can be processed.

### 2.3. Isolation, Identification, and Serotyping of *Salmonella* Isolates

The cloacal swabs were pre-enriched in buffered peptone water (BPW Oxoid, Biolab, South Africa) at 37°C for 24 hours. A loopful of the bacterial cells in buffered peptone water was streaked onto xylose-lysine-deoxycholate agar (Merck, Wadeville, South Africa) and Brilliant Green agar (Scharlau Chemie S.A. Barcelona, Spain). The streaked plates were then incubated at 37°C for 24 hours. The colonies were examined for their morphological appearance on the plate (colonies with or without black centers, colorless, or opaque-white colonies surrounded by pink or red zones on XLD). The suspected *Salmonella* spp. colonies, those with glossy large black centers or almost black colonies, were examined for pure culture isolation on BGA. Between three and five colonies were selected and purified on nutrient agar (Merck, Wadeville, South Africa) and incubated at 37°C for 18 to 24 hours.

### 2.4. DNA Extraction and Molecular Identification of *Salmonella* Serovars Using *invA* Gene

The bacterial genomic DNA was extracted using a genomic DNA extraction kit (Invitrogen, USA) from pure cultures. A NanoDrop spectrophotometer was used to measure the DNA concentrations. For the *invA* gene, PCR was carried out using the forward (GTG AAA TTA TCG CCA CGT TCG GGC AA) and reverse (TCA TCG CAC CGT CAA AGG AAC C) oligonucleotide primers with a reaction volume of 25 *μ*L, containing: 8.5 *μ*L nuclease-free water, 12.5 *μ*L PCR Master Mix, 2 *μ*L template DNA, and 1 *μ*L of each primer utilizing an Engine T100 ThermalTM cycler (BioRad, Singapore). The thermal cycling conditions included an initial step of denaturation at 94°C for 5 minutes, then 30 cycles of denaturation at 94°C for 45 seconds, annealing at 58°C for 45 seconds, and extension at 72°C for 70 minutes, followed by a single, concluding extension step at 72°C for 7 minutes [13].

### 2.5. Identification of *Salmonella* Species Using 16S rRNA

All the positive samples for *inv*A were subjected to 16S rRNA for sequencing. The bacterial universal primers (27F: AGA GTT TGA TCM TGG CTC AG and 1492R: GGT TAC CTT GTT ACG ACT T) targeting the 16S rRNA gene segment were used for molecular identification using PCR. The PCR conditions were as follows: initial denaturation step at 96°C for 4 minutes, followed by 30 cycles of denaturation at 94°C for 30 seconds, annealing at 57°C for 30 seconds, and extension at 72°C for 1 minute, and finally, a single and final extension step at 72°C for 10 minutes [22].

### 2.6. Sequencing of PCR Amplicons

The PCR products were sequenced at Inqaba Biotechnical Industries (Pty) Ltd., Pretoria, South Africa. The FintchTV [23] was used to edit the base pairs of the sequence chromatograms. Sequence identity was evaluated using the nucleotide Basic Local Alignment Search Tool nucleotide (BLASTn) on the NCBI website (https://blast.ncbi.nlm.nih.gov/Blast.cg). The generated *16S* rRNA gene sequences were submitted to the GenBank database and assigned with the accession numbers as follows: OP683334–OP683363.

### 2.7. Detection of Virulence Genes among *Salmonella* Serovars

All *Salmonella* spp. isolates were subjected to PCR screening for 17 (Supplementary Table S1) virulence genes [16, 24]. A PCR mix (25 *μ*L) was used, consisting of 8.5 *μ*L nuclease-free water, 12.5 *μ*L PCR, 2X DreamTaq Green Master Mix (Thermo-Fisher Scientific, South Africa), 2 *μ*L template DNA, and 1 *μ*L of each primer. The following PCR parameters were applied: 94°C for 5 minutes, 30 cycles of 94°C for 45 seconds, annealing temperatures (for each gene as shown in Supplementary Table S1) for 45 seconds, and 72°C for 1 minute; and 72°C for 10 minutes.

### 2.8. Antimicrobial Susceptibility Testing

Based on the guidelines of the Clinical and Laboratory Standards Institute (CLSI 2023) [25], *Salmonella* isolates were tested for their antimicrobial susceptibility to 13 different antimicrobial agents using the Kirby–Bauer disc diffusion method on Mueller–Hinton Agar (Oxoid Ltd., Basingstoke, UK). Antibiotics used in this study were streptomycin (30 *μ*g), ciprofloxacin (5 *μ*g), nalidixic acid (30 *μ*g), gentamicin (10 *μ*g), and kanamycin (30 *μ*g). Resistance to two or more antimicrobials of different classes was considered to be multidrug-resistant (MDR) [26].

### 2.9. Detection of Antibiotic Resistance Genes

All the *Salmonella* spp. were tested for the presence of quinolone (*qnrA*, *qnrS*, *parC*, and *aac*(*6*′)*-Ib-cr*) and aminoglycoside (*strA*, *strB*, and *aac*(*6*′)-*Ib*) resistance genes [27–29]. Antibiotic resistance genes were detected using the primers and annealing temperatures as shown in Supplementary Table S2.

## 3. Results

### 3.1. Occurrence of *Salmonella* Serovars in Reptiles Using *invA* and 16S rRNA

A total of 19 samples were collected from lizards (*n* = 6), snakes (*n* = 3), chameleons (*n* = 7), and tortoises (*n* = 3). From these, a total of 30 *Salmonella* spp. isolates were recovered from the various reptile species (Table 1). Based on nucleotide BLAST results of 16S rRNA sequences detected, *Salmonella* serovars/species were *S*. Salamae (*n* = 9; 30%), *S*. *enterica* (*n* = 5; 16.7%), *S*. Typhimurium (*n* = 4; 13.3%), *S*. Indiana (*n* = 4; 13.3%), and one for *Salmonella enterica* subsp. *enterica* serovar Abony, *S. enterica* subsp. *enterica* serovar Houtenae, *S. enterica* subsp. *enterica* serovar Waycross, *S. enterica* subsp. *enterica* serovar Typhi, *S. enterica* subsp. *enterica* serovar Kentucky, *S. enterica* subsp. *enterica* serovar Newlands, *S. enterica* subsp. *enterica* serovar Worthington, and *S. enterica* subsp. *enterica* serovar Paratyphi C.

### 3.2. Detection Rate and Distribution of Virulence Genes in Various Serotypes

**Figure 1:**
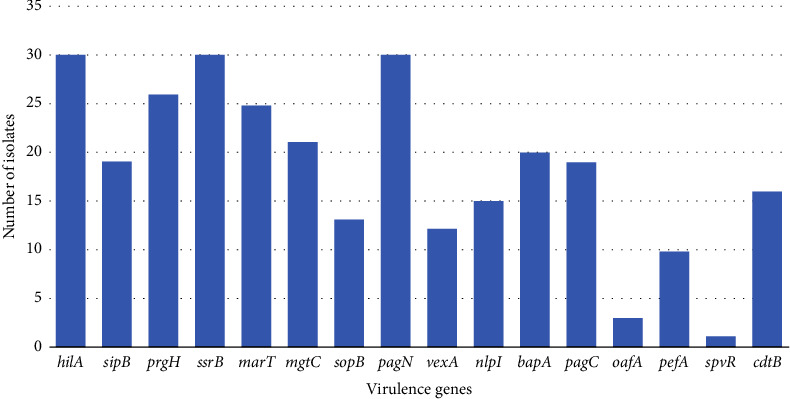
Distribution of virulence genes in different *Salmonella* serovars recovered from reptiles in South Africa.

**Figure 2:**
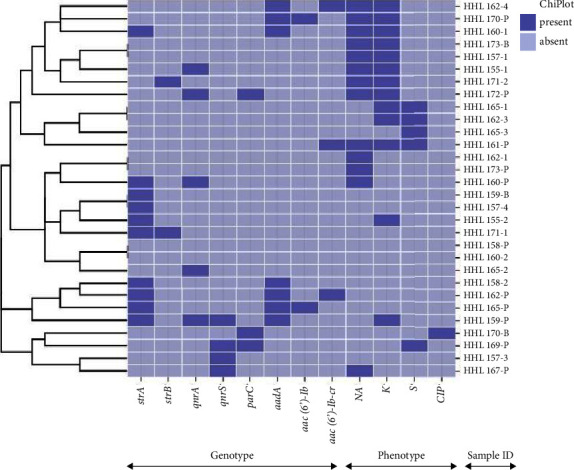
Heatmap showing the clustering of the antibiotic resistance profiles in the *Salmonella* isolates. Light blue and dark blue indicate the absence and presence of antibiotic and resistance genes, respectively, (https://www.chiPlot.online/#9 (accessed on 17 June 2023)).

A total of 30 *Salmonella* spp. isolates harbored either one or more different virulence genes investigated in this study, with sixteen out of seventeen virulence genes detected in this study (Figure 1). The distribution of virulence genes among each *Salmonella* isolate is shown on the heatmap (Figure 2). The majority of these isolates harbored the following genes; *pag*N (*n* = 30; 100%), *hil*A (*n* = 29; 96.7%), *ssr*B (*n* = 29; 96.7%), *prg*H (*n* = 26; 86.7%), *mar*T (*n* = 26; 86.7%), *mgt*C (*n* = 22; 73.3%), *bap*A (*n* = 21; 70%), *pag*C (*n* = 20; 66.7%), *sip*B (*n* = 19; 63.3%), *cdt*B (*n* = 17; 56.7%), *vex*A (*n* = 12; 40%), *nlp*I (*n* = 14; 46.7%), *pef*A (*n* = 9; 30%), *oaf*A (*n* = 2; 6.7%), *spv*R (*n* = 2; 6.7%), and *sop*B (*n* = 1; 3.3%). The *spv*B gene was not detected in any of the 30 isolates.

### 3.3. Antibiotic Susceptibility and Resistant Genes of *Salmonella* Isolates

*Salmonella* isolates in this study had the highest antibiotic resistance rates against nalidixic acid (13; 43.3%) (95% CI: 0.25 ± 0.62), kanamycin (13; 43.3%) (95% CI: 0.25 ± 0.62), streptomycin (5; 16.7%) (95% CI: 0.03 ± 0.31), and ciprofloxacin (1; 3.3%) (95% CI: −0.03 ± 0.0.10) using antibiotic disk diffusion assays (DDA). All 30 (95% CI: 0 ± 0) *Salmonella* isolates were susceptible to gentamicin. Out of the 30 isolates, nine (30%) *Salmonella* serovars harbored more than one antibiotic resistance gene. The distribution of the antibiotic resistance genes for each *Salmonella* isolate is shown on the heatmap (Figure 2). PCR was carried out for *Salmonella* isolates to screen for eight antibiotic resistance genes (ARGs). Out of 30 *Salmonella* isolates, the prevalence of the ARGs: *strA*, *strB*, *qnrA*, *qnrS*, *parC*, *aadA*, *aac*(*6ˊ*)-*Ib*, and *aac(6ˊ)-Ib-cr* genes was 10; 33.3%, 2; 6.7%, 5; 16.7, 4; 13.3%, 3; 10%, 7; 23.3%, 2; 6.7%, and 3; 10%, respectively. Among *Salmonella* serovars strains, the presence of the quinolones (*qnr*A, *qnr*S, and *par*C) genes correlated with phenotypic susceptibility.

## 4. Discussion

Reptiles carry zoonotic pathogens that cause a variety of infectious diseases in both humans and other animals [6]. They are becoming increasingly appealing as pets and are popular attractions at wildlife education centers [20]. Although the clinical relevance of *Salmonella* infections in wild and captive reptiles is poorly understood, it is believed that the majority of infections results in an asymptomatic carrier condition and do not cause disease in reptiles [6]. *S. enterica* subsp. *enterica* serovar Houtenae has been associated with abdominal abscesses in a severely diseased captive African fat-tailed gecko [30].

Our study confirmed that reptiles are reservoirs of multiple *Salmonella* serovars. There were a number of *Salmonella* serovars detected in different reptiles that are of public health concern and included *S*. *enterica*, *S*. Typhimurium, *S*. Indiana, *S*. Houtenae, *S*. Waycross, *S*. Typhi *S*. Kentucky, *S*. Newlands, *S*. Worthington, and *S*. Paratyphi C [31–34].

From a host-reservoir perspective, chameleons (*Chamaeleo dilepis*) were the most frequently infected with *Salmonella* serovars, i.e., *S*. *enterica*, *S*. Indiana, *S*. Salamae, *S*. Typhi, and *S*. Kentucky. The prevalence rates of *Salmonella* serovars among chameleons, lizards, snakes, and tortoises were 36.8%, 31.6%, 15.8%, and 15.8%, respectively. These findings differ in terms of the frequency of *Salmonella* spp. occurrence in various sectors of captive reptiles in Europe. Higher (76.9%) prevalences of *Salmonella* spp. were recorded in pet snakes, lizards, and tortoises from Poland [5], 64.5% in snakes and lizards from Norwegian zoos [1], and 32.6% in domestic snakes, chameleons, and lizards from central Europe [35], 43.28% of the pet reptiles carried from Western Romania [36], and 50.0% of the lizard from Fernando de Noronha Archipelago (Brazil) [37]. The current study is one of the few studies to isolate *Salmonella* serovars from wild reptiles.

The majority of salmonellosis illnesses are associated with a wide range of serotypes of *S*. *enterica* subsp. *enterica* (I) and are primarily transmitted through tainted food and water [30, 38–40]. In some parts of the world, pet reptiles provide a significant source of protein for human populations, and in so doing, a transmission route for *Salmonella* is established. All reptiles are exploited for human consumption, but turtles are heavily exploited, while crocodiles, snakes, and lizards may be important locally [41, 42]. Indeed, there have been numerous reports of reptile-associated salmonellosis in humans, especially in children [20, 43, 44].

*Salmonella* pathogenicity island 1 is essential for the interaction between *Salmonella* and host cells. *Salmonella* invades epithelial cells through SPI-1 (44). Two SPI-1 genes that encode components of the SPI-1 T3SS apparatus, *invF* and *sicA*, are directly regulated by the *OmpR*/*ToxR* transcriptional regulator HilA [45, 46]. Moreover, enterocolitis and human intestinal epithelial cell invasion may be influenced by the regulation of virulence factors including *HilA*, *invA*, and SPI-1 effectors such as *SipA* and *SopABD* [47, 48].

*Salmonella's* intracellular pathogenicity cycle begins with the invasion of intestinal epithelial cells, controlled by the *invA* gene [49]. *Salmonella*-specific gene sequences encode the *InvA* protein that is essential for gut epithelial invasion [50]. The results showed that all *Salmonella* isolates tested positive for the *invA* gene. This is in agreement with the findings of previous studies (12, 13, 21, 22, 37, and 49). It is not surprising because *InvA* is used for molecular identification of these *Salmonella* isolates [51]. Virulence gene profiles showed that all the *Salmonella* serovars isolated in this study were positive for *pag*N, *hil*A, *ssr*B, *prg*H, and *mar*T (100%), (96.7%), (96.7%), (86.7%), and (86.7%), respectively. Similar genes were detected in *Salmonella* species isolated from retail beef samples in selected KwaZulu-Natal municipality areas and in livestock production systems (cattle, sheep, goats, pigs, ducks, and chickens) in the Eastern Cape and KwaZulu-Natal provinces of South Africa [52, 53].

Virulence plasmid operons (*spvRABCD*) are expressed by intracellular environments in host cells and are involved in survival, intracellular growth, and macrophage death [54, 55]. The *spv*R gene was detected in one (3.3%) sample. This observation was different from the findings of a study conducted by Derakhshandeh et al. [56] on humans, where they reported that the prevalence of *spv*B, *spv*C, and *spv*R genes was 26 (43.3%), 44 (73.3%), and 28 (46.6%), respectively. The study on humans and animals reported in 2008 by Amini et al. [57] showed that the *spv*B and *spv*C genes were detected in 90% of the isolates. In the current study, the *spv*B gene was not detected in any of the 30 isolates. In Burkina Faso, Nikiema et al. [58] detected *spvR* and *spvC* genes at 36.8% and 48.1%, respectively, from 106 *Salmonella* isolates (77 human stools and 14 sandwiches). The *spvC* gene resides on plasmids and plays an important role in adhesion and systemic infection of host cells [59]. The *SipC* protein also targets F-actin, which is critical for the internalization and invasion of pathogens [50]. In consideration of the low level of detection of the *spv* gene in wild reptiles, there is a need to expand the surveillance to a broader host range over a larger geographical area.

Of the 17 virulence genes screened in this study, 13 are located on *Salmonella* pathogenicity islands (SPIs). All *Salmonella* isolates in this study exhibited high detection rates for virulence genes located on the SPIs, indicating the genes were widely distributed. The SPI-1 genes *sip, hil*, and *prg* encode regulators that produce T3SS effector proteins, assist in *Salmonella* colonization and invasion of intestinal epithelial cells, and can trigger macrophage necrosis and inflammatory responses [16].

Several researchers have recently reported the presence of antibiotic residues in reptiles and antibiotic-resistant bacteria [5–7, 60]. However, drug resistance in reptiles is relatively uncommon in reptile-associated *Salmonella* [60]. Although the prevalence of antimicrobial resistance was not very high in this study, *S*. Worthington had the widest range of antibiotic resistance (60%). High antibiotic resistance prevalence was observed for nalidixic acid (43.3%) and kanamycin (43.3%). In comparison to *Salmonella* isolates in water samples in the Philippines, resistance to kanamycin was higher at 75.4% [61]. On the other hand, there is a reported high (95.4%) nalidixic acid resistance by *Salmonella* isolates obtained from broiler and layer chicken farms [62]. Thirty-three isolates (33.3%) of *Salmonella* serovars were resistant to at least one antimicrobial drug. Similar findings were reported in studies involving *Salmonella* serovars isolated from reptiles from Taiwan, Trinidad, and Malaysia and their sensitivity to aminoglycosides and quinolones [7, 63, 64]. In the same study by Chen et al. [7], as well as a study from Lithuania, *Salmonella* serovars isolates from reptiles most frequently displayed resistance to streptomycin and tetracycline [6, 7], and in a study from Poland, the highest antibiotic resistance was detected against streptomycin [20]. In a study conducted by Dégi et al. [65] in Romania, *Salmonella* serovars isolated from reptiles were resistant to ceftriaxone, ciprofloxacin, vancomycin, cefoxitin, pristinamycin, ampicillin/sulbactam, and gentamicin. In contrast to our results, Abrahão et al. [37] have reported 13.3% of isolates from lizard resistant to colistin in Brazil. Given this growing evidence for antibiotic resistance, the importance of reptile-associated *Salmonella* spp. infections to medical research and public health should not be overlooked.

*Salmonella enterica* subsp. *enterica* serovar isolates from this study were resistant to aminoglycosides and quinolone classes of antibiotics. The same antibiotic resistance gene profiles were detected in *Salmonella* serovars isolated from other animals, including commercial chickens, as well as humans in South Africa [66–70]. Similar antibiotic resistance genes (*strA*, *strB*, and *aadA*) were also detected in reptiles in Poland [20]. Both *strA* and *strB* genes encode aminoglycoside-3″-phosphotransferase (APH(3″)-Ib) and aminoglycoside-6-phosphotransferase (APH(6)-Id) proteins that confer streptomycin resistance, respectively [71].

Strains typically pose a high risk for the spread of resistance genes to other microbiota as well as for the treatment of infections [72]. Antimicrobial resistance is rapidly developing and spreading due to interactions between human, animal, and environmental factors [67]. There was a correlation between the presence of the quinolones (*qnrA*, *qnrS*, and *parC*) genes and the phenotypic susceptibility of the *Salmonella* serovar strains. Fluoroquinolones are widely used in veterinary practice, but no data involving the incidence of resistance exist [69]. Further research is needed to investigate the possible relationships of microorganism transfer between reptiles and other hosts.

### 4.1. Limitation of the Study

The main drawback of dealing with wild reptiles is how difficult it is to obtain more specimen samples. When it comes to reptile research and surveys, Africa is far less advanced than other continents [73]. Areas where reptiles occur in South Africa are usually remote and challenging to work with and sample in, which creates a sampling bias at times, which makes it very difficult for the collection of wild datasets [74]. In Barends et al. [74] work in what is irrefutably the most famous park or reserve in South Africa (Kruger National Park) to examine reptile species presence within the 1 km resolution, 92% of KNP would be considered “data deficient” for reptile occurrence. As mentioned in our methods section, Timbavati borders KNP and has the same “big five” (lion, leopard, rhino, buffalo, and elephant) dangers for field researchers in terms of sampling [19, 74].

## 5. Conclusions

According to our knowledge, this is the first study reporting on the occurrence, antibiotic resistance, and virulence profiles of *Salmonella* serovars from wild reptiles in South Africa. Chameleons had the highest infection rates for *Salmonella* serovars, followed by lizards, snakes, and turtles. Reptiles can serve as a reservoir for pathogenic bacteria such as *Salmonella*; hence, precautions should be taken when caring for and transporting them, as well as when keeping them in close contact with other animals. There is optimism for effective antibiotic therapy in the case of infection due to the low level of drug resistance of the reptile *Salmonella* serovars detected in the current study. The findings highlight the need for educational efforts aimed at reducing reptile-related infections. As previous literature cited in this study has mentioned that the prevalence of *Salmonella* appears higher in captive reptiles elsewhere in the world, we suggest the next logical step would be an investigation of *Salmonella* prevalence in captive reptiles in the South African pet trade, and with a particular focus on nonnative popular species.

## Figures and Tables

**Table 1:** *Salmonella* spp. serovars identification from different reptile species.

Reptile group	Species (*n*)	*Salmonella* serovars	Number of *Salmonella*isolates
Lizard	*Metabosorus validis* (2)	*S*. Salamae, *S*. Houtenae, and *S*. Salamae	10/30 (33%)
	*Chondrodactylus turneri* (2)	*S*. Waycross and *S*. Indiana	
	*Trachylepis striata* (2)	*S*. Typhimurium, *S*. Salamae, and *S*. Salamae	

Snake	*Philothamnus semivariegatus* (2)	*S*. Worthington	3/30 (10%)
	*Bitis arietans* (1)	*S.* Typhimurium and S. Indiana	
	*Dispholidus typus* (1)		

Tortoise	*Stigmochelys pardalis* (3)	*S*. Newlands and *S. enterica*	2/30 (6.7%)

Chameleon	*Chamaeleo dilepis* (7)	*S*. *enterica*, *S*. Indiana, *S*. Salamae, *S.* Typhi, and *S*. Kentucky	15/30 (50%)

## Data Availability

The data that support the findings of this study are made available from the corresponding author upon reasonable request.

## Supplementary Materials

Supplementary Materials Supplementary Table S1. Oligonucleotide primers used for detection of virulence associated genes of *Salmonella* isolates. Supplementary Table S2. List of antibiotic resistance genes primers and conditions used in this study.
