# Supplementary material for: Occurrence, Antimicrobial Resistance, and Virulence Profiles of Salmonella Serovars Isolated from Wild Reptiles in South Africa
Source: Int J Microbiol. 2024 Jan 5;2024:5213895. doi: 10.1155/2024/5213895 (PMC10787053; doi:10.1155/2024/5213895)
Supplement: Supplementary Materials — Supplementary Table S1. Oligonucleotide primers used for detection of virulence associated genes of Salmonella isolates. Supplementary Table S2. List of antibiotic resistance genes primers and conditions used in this study. [file 5213895.f1.docx]

**Occurrence, antimicrobial resistance and virulence profiles of *Salmonella* serovars isolated from wild reptiles in South Africa**

Lungile N. Mlangeni^1^, Tsepo Ramatla^1,2^*, Kgaugelo E. Lekota^1^, Cormac Price^1^, Oriel Thekisoe^1^, Che Weldon^1^

^1^Unit for Environmental Sciences and Management, North-West University, Potchefstroom, 2531, South Africa

^2^Gastrointestinal Research Unit, Department of Surgery, School of Clinical Medicine, University of the Free State, Bloemfontein 9300, South Africa

Correspondence should be addressed to Tsepo Ramatla, Email: [ra21205450@gmail.com](mailto:ra21205450@gmail.com)

**Supplementary Table S1.** Oligonucleotide primers used for detection of virulence associated genes of *Salmonella* isolates.

| **Gene** | **Location** | **Primer name** | **Primer sequence (5′-3′)** | **Length (bp)** | **Annealing temp (°C)** | **References** |
| --- | --- | --- | --- | --- | --- | --- |
| *hilA* | SPI-1 | hilA-F  hilA-R | GACAGAGCTGGACCACAATAAGACA  GAGCGTAATTCATCGCCTAAAC | 312 | 55°C | [1] |
| *sipB* | SPI-1 | sipB-F  sipB-R | GGACGCCGCCCGGGAAAAACTCTC  ACACTCCCGTCGCCGCCTTCACAA | 875 | 66.5°C | [2] |
| *prgH* | SPI-1 | prgH-F  prgH-R | CTTCAGGYCAACTCCCTGATATAC  CCCTTGAGCCAGTCATCTTT | 961 | 55°C | [1] |
| *ssrB* | SPI-2 | ssrB-F  ssrB-R | CTCATTCTTCGGGCACAGTTA  CCTTATTACCCTGGCCTCATTT | 558 | 55°C | [1] |
| *marT* | SPI-3 | marT- F  marT-R | CGTCGTCTCACAACAAACATTC  CTGACAAATCAATGCCGTAACC | 556 | 55°C | [1] |
| *mgtC* | SPI-3 | mgtC-F  mgtC-R | AAAGACAATGGCGTCAACGTATGG  TTCTTTATAGCCCTGTTCCTGAGC | 500 | 65°C | [1] |
| *sopB* | SPI-5 | sopB-F  sopB-R | TCACTAAAAACCCAGGAGGCTTTT  CGCCATCTTTATTGCGGATTTTTA | 1000 | 65°C | [1] |
| *pagN* | SPI-6 | pagN-F  pagN-R | TTCCAGCTTCCAGTACGTTTAG  GCCTTTGTGTCTGCATCATAAG | 440 | 55°C | [1] |
| *vexA* | SPI-7 | vexA-F  vexA-R | AAACTAAGCGCTCCCGATAC  CAGTCGCGCAGTGAAATAATG | 504 | 55°C | [1] |
| *nlpI* | SPI-8 | nlpI-F  nlpI-R | AGTCTTGGTTTGAGGGCATTAG  TTCTTTCGCCTGCTTCTCATTA | 333 | 55°C | [1] |
| *bapA* | SPI-9 | bapA-F  bapA-R | TAAGCGTCGGACTTGGAATG  CGTTCTTCAGCGTGTAGGTATAG | 543 | 55°C | [1] |
| *pagC* | SPI-11 | pagC-F  pagC-R | CGCCTTTTCCGTGGGGTATGC  GAAGCCGTTTATTTTTGTAGAGGAGATGTT | 454 | 66.5°C | [2] |
| *oafA* | SPI-12 | oafA-F  oafA-R | CGAGTGACTGGAACCAAAGA  CAAGCATAGAGCCAGAGTAGAG | 510 | 55°C | [1] |
| *spvB* | Plasmid | spvB-F  spvB-R | CTATCAGCCCCGCACGGAGAGCAGTTTTTA  GGAGGAGGCGGTGGCGGTGGCATCATA | 717 | 66.5°C | [1] |
| *pefA* | Plasmid | pefA-F  pefA-R | GCGCCGCTCAGCCGAACCAG  GCAGCAGAAGCCCAGGAAACAGTG | 157 | 66.5°C | [1] |
| *spvR* | Plasmid | spvR-F  spvR-R | CCGCTGAGCAGGGTTATTT  CTTGGTCGGGTAATACAAGGAG | 723 | 55°C | [1] |
| *cdtB* | Genome | cdtB-F  cdtB-R | ACAACTGTCGCATCTCGCCCCGTCATT  CAATTTGCGTGGGTTCTGTAGGTGCGAGT | 268 | 66.5°C | [1] |

**Supplementary Table S2.** List of antibiotic resistance genes primers and conditions used in this study.

| **Class and Target gene** | **Primer** | **Primer sequence (5´ → 3´)** | **Amplicon size (bp)** | **Annealing temp (°C)** | **References** |
| --- | --- | --- | --- | --- | --- |
| **Quinolone** |  |  |  |  |  |
| *qnr*A | qnrA-F  qnrA-R | ATTTCTCACGCCAGGATTTG  GAGATTGGCATTGCTCCAGT | 413 | 56 | [3] |
| *qnrS* | qnrS-F  qnrS-R | CCCCATGCCCGAAGTTATCA  ACTGCTTGGAGTGTGTTGGT | 457 |  | [3] |
| *par*C | parC-F  parC-R | GCCTAAACAACGCACGGAAA  TGACACGGGAGGTAACCAGA | 432 | 59 | [3] |
| *aac(6**ˊ)-Ib-cr* | aac(6ˊ)-Ib-cr-F  aac(6ˊ)-Ib-cr-R | ATATGCGGATCCAATGAGCAACGCAAAAACAAAGTTAG  ATAGCGAATTCTTAGGCATCACTGCGTGTTCGCT | 544 | 66 | [4] |
| **Aminoglycoside** |  |  |  |  |  |
| *str*A | strA-F  strA-R | CTTGGTGATAACGGCAATTC  CCAATCGCAGATAGAAGGC | 548 | 55 | [5] |
| *str*B | strB-F  strB-R | ATCGTCAAGGGATTGAAACC  GGATCGTAGAACATATTGGC | 509 | 56 | [5] |
| *aac(*6ˊ)*-*Ib | aac(6ˊ)-Ib-F  aac(6ˊ)-Ib-R | TATGAGTGGCTAAATCGAT  CCCGCTTTCTCGTAGCA | 395 | 55 | [4] |

**References**

1. M. Yue, X. Li, D.Liu, and X. Hu, “Serotypes, antibiotic resistance, and virulence genes of *Salmonella* in children with diarrhea”. *Journal of Clinical Laboratory Analysis*, vol. 34, pp. e23525, 2020.
2. H. P. Xiong, M. Tang, and H. P. Zhang, “The preliminary study about distribution features of *Salmonella* virulence genes in Nantong city”. *Journal of Qiqihar Medical College*, vol, 30, no. 19, pp. 2355-2357, 2009.
3. J. Xu, Y. Xu, H. Wang, C. Guo, H. Qiu, Y. He, Y. Zhang, and X. Li, W, “Occurrence of antibiotics and antibiotic resistance genes in a sewage treatment plant and its effluent-receiving river”. *Chemosphere*, vol. 119, pp. 1379-1385, 2015.
4. S. F. Huang, W. Dai, S. Sun, X. J. Zhang, and L. P. Zhang, “Prevalence of plasmid-mediated quinolone resistance and aminoglycoside resistance seterminats among carbapeneme non-susceptible”. *Enterobacter cloacae*. *Plos One*, vol. 7, pp. e47636, 2012.

L. Ouoba, V. Lei, and L. B. Jensen, “Resistance of potential probiotic lactic acid bacteria and bifidobacteria of African and European origin to antimicrobials: Determination and transferability of the resistance genes to other bacteria”. *International Journal of Food Microbiology*, vol. 121, pp. 217 – 224, 2008
